# Susceptibility and Resistance of SARS-CoV-2 Variants to LCB1 and Its Multivalent Derivatives

**DOI:** 10.3390/v16010036

**Published:** 2023-12-25

**Authors:** Hongliang Jin, Yani Gong, Lin Cheng, Yuanmei Zhu, Zheng Zhang, Yuxian He

**Affiliations:** 1 NHC Key Laboratory of Systems Biology of Pathogens, Institute of Pathogen Biology, Chinese Academy of Medical Sciences and Peking Union Medical College, Beijing 102600, China; jinhongliang588@163.com (H.J.); gongyani@ipbcams.ac.cn (Y.G.); zhuym@ipbcams.ac.cn (Y.Z.); 2 Institute of Hepatology, National Clinical Research Center for Infectious Disease, Shenzhen Third People’s Hospital, The Second Affiliated Hospital, School of Medicine, Southern University of Science and Technology, Shenzhen 518112, China; chenglin252@163.com

**Keywords:** SARS-CoV-2, receptor binding motif (RBM), LCB1, multivalent inhibitor, drug resistance

## Abstract

LCB1 is a computationally designed three-helix miniprotein that precisely targets the spike (S) receptor-binding motif (RBM) of SARS-CoV-2, exhibiting remarkable antiviral efficacy; however, emerging SARS-CoV-2 variants could substantially compromise its neutralization effectiveness. In this study, we constructed two multivalent LCB1 fusion proteins termed LCB1T and LCB1T-Fc, and characterized their potency in inhibiting SARS-CoV-2 pseudovirus and authentic virus in vitro. In the inhibition of various SARS-CoV-2 variants, the two LCB1 fusion proteins exhibited markedly improved inhibitory activities compared to LCB1 as anticipated; however, it was observed that relative to the D614G mutation hosting variant, the variants Delta, Lambda, and Omicron BQ.1.1, XBB, XBB.1.5, and EG.5.1 caused various degrees of resistance to the two fusion proteins’ inhibition, with XBB, XBB.1.5, and EG.5.1 variants showing high-level resistance. Moreover, we demonstrated that bat coronavirus RaTG13 and pangolin coronavirus PCoV-GD/PCoV-GX were highly sensitive to two LCB1 fusion proteins, but not LCB1, inhibition. Importantly, our findings revealed a notable decrease in the blocking capacity of the multivalent LCB1 inhibitor on the interaction between the virus’s RBD/S and the cell receptor ACE2 when confronted with the XBB variant compared to WT and the Omicron BA.1 variant. In conclusion, our studies provide valuable insights into the antiviral profiling of multivalent LCB1 inhibitors and offer a promising avenue for the development of novel broad-spectrum antiviral therapeutics.

## 1. Introduction

Severe acute respiratory syndrome coronavirus 2 (SARS-CoV-2), the novel virus that causes the COVID-19 pandemic, belongs to a family of highly contagious betacoronaviruses. Since its emergence in late 2019, SARS-CoV-2 has led to more than 760 million confirmed cases and over 6.9 million reported deaths worldwide (https://covid19.who.int/table (3 September 2023)). While the rapid development of vaccines has notably curtailed severity and mortality, SARS-CoV-2 continues to undergo evolution and mutation, resulting in the continual emergence of novel variants. For example, the Omicron variant, which emerged in November 2021, has given rise to numerous lineages that have rapidly disseminated globally, posing challenges to the existing vaccine and treatment strategies [1,2,3,4,5].

Spike (S) protein of SARS-CoV-2 virus forms a trimer in its functional state, and it plays an essential role in viral attachment, fusion, and entry, and thus it has been used as a primary molecular target to develop prophylactics and therapeutics against the ongoing viral spread [6,7,8]. In particular, the receptor-binding domain (RBD) within the S protein is accountable for binding to the human cell’s angiotensin-converting enzyme 2 (ACE2) receptor, which serves as the initiator of the virus infection process [7,9,10]. The homotrimeric RBD of the SARS-CoV-2 S protein can switch between “up” and “down” conformations. When RBD is in the “up” conformation, its receptor-binding motif (RBM) region is exposed and ACE2 is able to bind to it [11]. Consequently, disrupting the RBD-ACE2 interaction is a promising therapeutic approach for preventing virus entry. Based on the structure of the two N-terminal α-helices of ACE2, Cao and coworkers generated a collection of miniproteins through computational structure design, which could bind to RBD, impeding its interaction with ACE2 and effectively obstructing the infection process. Functional screens showed that a lead miniprotein, termed LCB1, has 56 amino acids and three distinct α-helices, and could structurally contact the RBM of RBD and inhibit SARS-CoV-2 infection with remarkable potency [12]. Given the encouraging characteristics of LCB1, the same group also demonstrated that the modified LCB1 inhibitors displayed prevention and treatment potential in human ACE2 (hACE2)-expressing transgenic mice against SARS-CoV-2 challenge [13]. Discouragingly, several studies unveiled that mutations present in the continuously emerging SARS-CoV-2 variants could significantly reduce the binding affinity and neutralizing effectiveness of LCB1 [13,14,15,16], thus challenging its potential as a candidate for drug development. Our results showed that five variants of concern (VOCs)—Alpha (B.1.1.7), Beta (B.1.351), Gamma (P.1), Delta (B.1.617.2), and Omicron (BA.1)—displayed different degrees of resistance to LCB1, of which, Beta, Gamma, and Omicron possessed a high level of resistance, while Alpha and Delta had medium-level resistance [16]. In response to the challenge posed by LCB1 resistance, we recently exploited the truncation and lipid modification approach to design several truncated LCB1-based lipopeptides, and demonstrated that they had greatly improved potency in inhibiting diverse LCB1-resistant viruses [17]. However, multiple variants and some RBD mutations still had moderate resistance to LCB1-based lipopeptides. Emerging studies indicate that increasing the valency of protein- and nanobody-based inhibitors is an effective strategy for augmenting the binding affinity for the target antigen and the neutralizing activity, including against SARS-CoV-2 [18,19,20,21,22,23,24,25]. In this study, we embarked on the construction of two multivalent LCB1 fusion proteins and characterized their potential against the continuously emerging SARS-CoV-2 variants.

## 2. Materials and Methods

### 2.1. Plasmids and Cell Lines

The plasmids expressing the S proteins of the SARS-CoV-2 WH-Hu-1 strain (WT) and VOCs (Alpha, Beta, Gamma, Delta, Lambda, and the Omicron sublineages BA.1, BA.2, BA.2.12.1, BA.4/5, BF.7, BQ.1.1, XBB, XBB.1.5, and EG.5.1) were generously provided by Linqi Zhang (Tsinghua University, Beijing, China) [26]. The plasmids expressing the S proteins of Guangdong and Guangxi pangolin-derived coronaviruses (PCoV-GD and PCoV-GX) and bat-derived coronavirus RaTG13 were kindly provided by Youchun Wang (National Institutes for Food and Drug Control, Beijing, China) [27]. The plasmid expressing the S protein of Omicron sublineage BA.2.13 was constructed and maintained in our laboratory. The LCB1T construct comprising three-tandem LCB1 with two (G4S)_5_ linkers and a C-terminal His tag, and the LCB1T-Fc construct including three-tandem LCB1 with two (G4S)_5_ linkers and a C-terminal IgG1 Fc domain were synthesized by Tsingke Biotechnology Co. (Tianjin, China). The two constructs were inserted into the eukaryotic cell expression vector pcDNA3.4 between the Xba I and Age I sites. HEK293T cells and Vero E6 cells were obtained from ATCC. 293T/ACE2 cells (hACE2, stably expressed by HEK293T) were produced and preserved in our laboratory [28]. All cells were maintained in Dulbecco’s minimal essential medium (DMEM, Gibco) supplementing with 10% fetal bovine serum (FBS), 1% penicillin–streptomycin, and 1 mM sodium pyruvate at 37 °C in a humidified atmosphere of 5% CO_2_. 

### 2.2. Expression and Purification of Multivalent LCB1 Inhibitors

To generate multivalent LCB1 inhibitors, HEK293T cells were transfected with 75 μg of plasmid in each 150 mm dish using a linear PEI transfection reagent. After 6 h, the culture medium was changed with fresh complete DMEM containing 1% FBS. After an additional 48 h of incubation at 37 °C, supernatants were collected via centrifugation at 10,000 rpm for 5 min and purified using nickel-chelated affinity column chromatography, according to the manufacturer’s protocol (QIAGEN GmbH, Hilden, Germany). Purified LCB1 fusion protein buffer was exchanged with phosphate-buffered saline (PBS, pH 7.4) and concentrated using an amicon ultra-15 10 k centrifugal filter device (Millipore, Billerica, MA, USA) and stored in PBS at −80 °C. To check the purity and molecular weight of purified LCB1 fusion proteins, the protein samples along with control LCB1 miniprotein were analyzed with NuPAGE 4–12% gradient Bis-Tris gel followed by Coomassie blue staining.

### 2.3. Pseudovirus Inhibition Assay 

The inhibitory efficacies of multivalent inhibitors on a variety of sarbecoviruses were determined through a single-cycle infection assay as described previously [29]. Briefly, the corresponding pseudoviruses (PsVs) of SARS-CoV-2, SARS-CoV, RaTG13, and PCoV were generated by cotransfecting HEK293T cells with a lentivirus backbone plasmid (pNL4-3.Luc.R-E-) and a viral S protein-expressing plasmid, including WT; D614G; Alpha; Beta; Gamma; Delta; Lambda; BA.1; BA.2; BA.2.12.1; BA.2.13; BA.4/5; BF.7; BQ.1.1; XBB; XBB.1.5; EG.5.1; SARS-CoV; RaTG13; PCoV-GD; and PCoV-GX, using a linear PEI transfection reagent. After 48 h of incubation, the pseudovirus-containing supernatants were collected through centrifugation at 4000 rpm for 10 min and stored in aliquots in a freezer at −80 °C. To assess the inhibitory activities of multivalent LCB1 fusion proteins, 50 μL of inhibitors was 3-fold serially diluted with DMEM containing 10% FBS and mixed with 50 μL of pseudovirus. At 1 h postincubation, the inhibitor–virus mixture was then added to 293T/ACE2 (3 × 10^4^ cells per well) for infection and incubated for an additional 48 h. The relative light units (RLUs) of cells were measured with a luciferase assay system and a luminescence counter (Promega, Madison, WI, USA). The percent inhibition of pseudovirus infection and 50% inhibitory concentration (IC50) of an inhibitor were calculated using GraphPad Prism software (GraphPad Software Inc., San Diego, CA, USA).

### 2.4. Live SARS-CoV-2 Virus Inhibition Assay

To measure the inhibitory activity of multivalent inhibitors against replicative SARS-CoV-2, a focus reduction neutralization test (FRNT) was conducted in a certified Biosafety level 3 laboratory as previously described with minor modifications [30]. In brief, serial 3-fold dilutions of LCB1T/LCB1T-Fc inhibitor were added to Vero E6 cells seeded in 96-well plates and incubated for 1 h at 37 °C. Then, 200 foci forming units of a live virus (WT, Delta, Omicron BA.2 or BA.4) were added to the culture wells and incubated for 1 h at 37 °C. After replacing the supernatants with medium containing 1.6% carboxymethylcellulose and 2% FBS, the plates were incubated at 37 °C for an additional 24 h. The cells were then fixed with 4% paraformaldehyde solution, permeabilized with Perm/Wash buffer (BD Biosciences, Franklin Lakes, NJ, USA) containing 0.1% Triton X-100, and incubated with HRP-conjugated anti-SARS-CoV-2 N protein antibody P301-F7. The reactions were developed using a KPL TrueBlue peroxidase substrate (Seracare Life Sciences Inc., Cambridge, MA, USA). The number of virus foci was quantified using an EliSpot reader (Cellular Technology Ltd., Cleveland, OH, USA). The percent inhibition of virus infection and 50% inhibitory concentration (IC_50_) of LCB1T and LCB1T-Fc were calculated using GraphPad Prism software (GraphPad Software 8.0).

### 2.5. Flow Cytometry Assay 

The blocking activity of multivalent inhibitor on the interaction of ACE2 and RBD/S proteins was determined using flow cytometry as described previously [29]. Briefly, 4 μg/mL of an RBD or S protein was prepared and mixed fully with serially diluted LCB1T and LCB1 inhibitor, respectively. After incubation for 1 h at 4 °C, the mixture was added to 5 × 10^5^ 293T/ACE2 cells for further incubation for 1 h at 4 °C. Then, the RBD- or S-binding cells were washed twice with FACS buffer (PBS solution with 0.5% bovine serum albumin and 2 mM EDTA) and stained with an APC-conjugated mouse anti-His tag antibody (BioLegend, San Diego, CA, USA) at 4 °C for 30 min. After two washes with FACS buffer, the stained cells were resuspended in 0.2 mL of FACS buffer and analyzed with a FACS CantoII instrument (Becton-Dickinson, Mountain View, CA, USA).

## 3. Results

### 3.1. Construction of Multivalent LCB1 Proteins with Improved Anti-SARS-CoV-2 Activity

**Figure 1:**
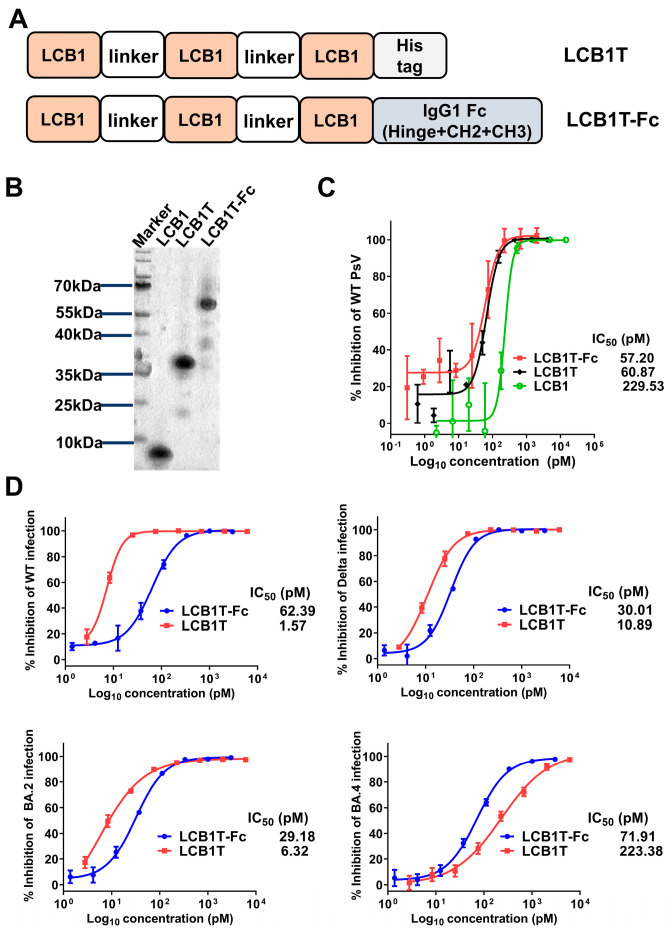
Construction of multivalent LCB1 proteins with improved anti-SARS-CoV-2 activity. (**A**) Schematic plot of design strategy of multivalent LCB1 proteins, where LCB1T represents three-tandem LCB1 repeats linked by a (G_4_S)5 linker, and where a C-terminus His tag is added. LCB1T-Fc was generated by fusing LCB1T with the Fc domain of IgG1. (**B**) The analysis of purity and size of recombinant multivalent proteins, as determined using SDS-PAGE. (**C**) Inhibition of recombinant proteins along with parental inhibitor on SARS-CoV-2 WT pseudovirus. (**D**) Inhibition of recombinant proteins on live SARS-CoV-2 WT, Delta, BA.2, and BA.4 strains, as determined in Vero E6 cells through a focus reduction neutralization test assay.

We previously found that a peptide-based LCB1 inhibitor showed dramatically reduced inhibitory activity against multiple SARS-CoV-2 variants [16]. To evaluate the potential of multivalency to increase the neutralization potency of LCB1, we first cloned a trivalent and its Fc-fused constructs as homotrimer and Fc fusion proteins, respectively (Figure 1A). The trivalent construct was prepared by connecting the LCB1 domains with five repeats of 15-residue linkers ((G_4_S)_5_) in a head-to-tail fashion. The human IgG1 Fc fragment was attached to the C terminal of the trivalent construct to generate the Fc-fused construct. Both the constructs were transiently expressed in HEK293T cells and purified from culture supernatants with a Ni Sepharose 6 Fast Flow Column. SDS-PAGE analysis revealed that the two multivalent LCB1 fusion proteins demonstrated high purities and expected sizes (Figure 1B).

We then evaluated the impact of valency on the ability of the LCB1 multivalent constructs to neutralize the SARS-CoV-2 WH-Hu-1 strain (WT) pseudovirus, as determined through a single-cycle infection assay. As shown in Figure 1C, compared to LCB1 template peptide, two fusion proteins exhibited similarly improved antiviral activity. Specifically, the trivalent-Fc and trivalent LCB1 fusion proteins displayed IC_50_ values of 57.20 pM and 60.87 pM, respectively, in inhibiting the WT pseudovirus. In comparison with the LCB1 template inhibitor, the two fusion proteins were about four-fold more potent in inhibiting the WT virus. Intriguingly, the addition of the Fc domain to the trivalent construct (trivalent-Fc) did not yield synergistic improvements in neutralization activity. 

To characterize whether the two fusion proteins were effective in inhibiting live SARS-CoV-2 strains, we next evaluated their inhibitory activity by applying four representative live SARS-CoV-2 strains in vitro—WT, Delta, BA.2, and BA.4—to infect Vero E6 target cells. As determined in a focus reduction neutralization test (FRNT), both fusion proteins could efficiently inhibit the four live SARS-CoV-2 at picomole concentrations, with IC_50_s of 29.18–71.91 pM for trivalent-Fc LCB1 and IC_50_s of 1.57–223.38 pM for trivalent LCB1 (Figure 1D). Comparatively, the trivalent-Fc LCB1 exhibited consistent neutralization activity against all four live viruses, whereas the trivalent LCB1 displayed IC_50_ values that were 4-fold higher against BA.2, 7-fold higher against Delta, and 142-fold higher against BA.4 strains, as compared to its IC_50_ against the WT strain.

### 3.2. Varied Sensitivities of Diverse SARS-CoV-2 Variants to Multivalent LCB1 Inhibitor

**Figure 2:**
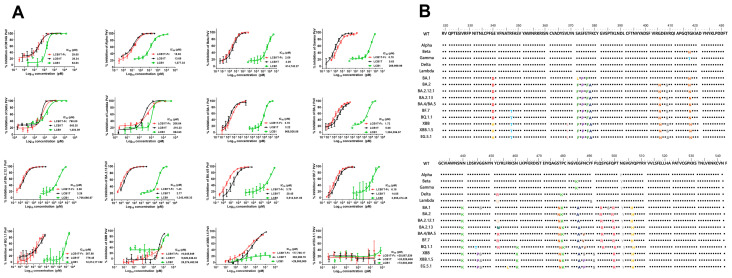
Inhibitory activities of LCB1-derived recombinant proteins against divergent SARS-CoV-2 variants. (**A**) The inhibitory activities of recombinant proteins along with the LCB1 control against numerous SARS-CoV-2 variants including Alpha, Beta, Gamma, Delta, Lambda, BA.1, BA.2, BA.2.12.1, BA.2.13, BA.4/5, BF.7, BQ.1.1, XBB, XBB.1.5, and EG.5.1 were determined on 293T/ACE2 cells through a pseudovirus-based single-cycle infection assay. (**B**) Different mutations were present in the RBDs of the different SARS-CoV-2 variants tested above. And different colors represent different amino acids.

In light of the pronounced resistance displayed by SARS-CoV-2 variants containing several RBD mutations to LCB1, we next assessed the potential of multivalency to enhance the resistance barrier of LCB1. To this end, a panel of pseudoviruses expressing SARS-CoV-2 spike protein of the divergent variants was prepared and used to evaluate the antiviral activity of the two fusion proteins, as determined via a single-cycle infection assay. As shown in Figure 2 and Table 1, as anticipated, all tested variant viruses, in comparison to the D614G variant, exhibited significant resistance to the LCB1 inhibitor, which was particularly notable in the case of Beta, Gamma, and various Omicron variants. For instance, compared to its IC_50_ on D614G, LCB1 exhibited an IC_50_ increase of at least 10,000-fold for multiple Omicron variants, and impressive 266,523.81-fold, at least 300,000-fold, and 700,000-fold increases in IC_50_s for the XBB, XBB.1.5, and EG.5.1 variants, respectively. In stark contrast, both LCB1 fusion proteins demonstrated substantially enhanced inhibitory activities against the majority of variant viruses, including Alpha, Beta, Gamma, BA.1, BA.2, BA.2.12.1, BA.2.13, BA.4/BA.5, and BF.7, whereas Delta, Lambda, and BQ.1.1 showed mild resistance to both LCB1 fusion proteins. An important observation pertains to the substantial reduction in potency of the two fusion proteins against the XBB, XBB.1.5, and EG.5.1 variants. Specifically, the trivalent-Fc and trivalent proteins exhibited IC_50_ values at least 200,000-fold and 318,498.46-fold higher, respectively, on XBB, 6119.97-fold and 13,489.06-fold higher, respectively, on XBB.1.5, and at least 700,000-fold and 800,000-fold higher, respectively, on EG.5.1, as compared to their IC_50_ values on D614G. These results highlighted, on the one hand, the availability of trivalent proteins as a tool to enhance inhibitor activity and, on the other hand, the robust resistance of the Omicron XBB and XBB.1.5 variants to the inhibitory effects exerted by the two multivalent LCB1 inhibitors.

### 3.3. High Sensitivity of Pangolin and Bat CoVs to Multivalent LCB1 Proteins

**Figure 3:**
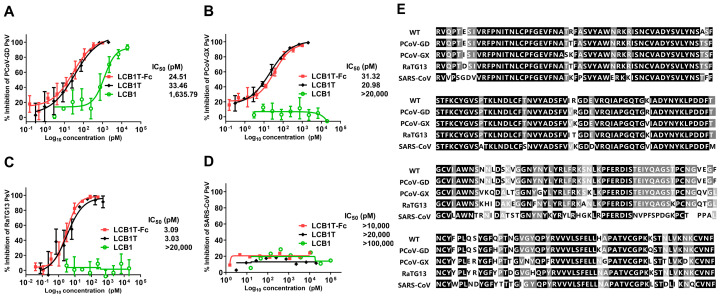
Inhibitory activities of LCB1-derived recombinant proteins and the LCB1 control inhibitor on SARS-CoV, Pangolin, and bat coronaviruses, as well as a multiple sequence alignment of the above RBDs. The inhibitory activities of recombinant proteins against PCoV-GD (**A**), PCoV-GX (**B**), bCoV-RaTG13 (**C**), and SARS-CoV (**D**) were measured on 293T/ACE2 cells in a pseudovirus-based single-cycle infection assay. (**E**) Multiple sequence alignment of the RBD from SARS-CoV-2 WT; PCoV-GD; PCoV-GX; RaTG13; and SARS-CoV.

Considering the strong relationship of SARS-CoV, pangolin, and bat coronaviruses (CoVs) to SARS-CoV-2, we were motivated to investigate whether the trivalent-Fc and trivalent proteins were highly effective in inhibiting these related viruses. To this end, the pseudoviruses expressing the S proteins of SARS-CoV, PCoV-GD, PCoV-GX, and bat CoV RaTG13 were generated and a single-cycle infection assay was similarly performed. As shown in Figure 3, PCoV-GD, PCoV-GX, and RaTG13 viruses were highly susceptible to the trivalent-Fc and trivalent LCB1 inhibition, whereas SARS-CoV displayed high resistance to the two fusion proteins’ inhibition, as anticipated. However, among the four viruses assessed, LCB1 only exhibited effective inhibition against the PCoV-GD virus, suggesting its closer genetic relationship with SARS-CoV-2. Our results underscored that CoVs closely related to SARS-CoV-2 are notably susceptible to the inhibitory influence of multivalent LCB1 proteins.

### 3.4. Omicron XBB Variant Greatly Impairs the Binding Affinity of Multivalent LCB1 Protein

**Figure 4:**
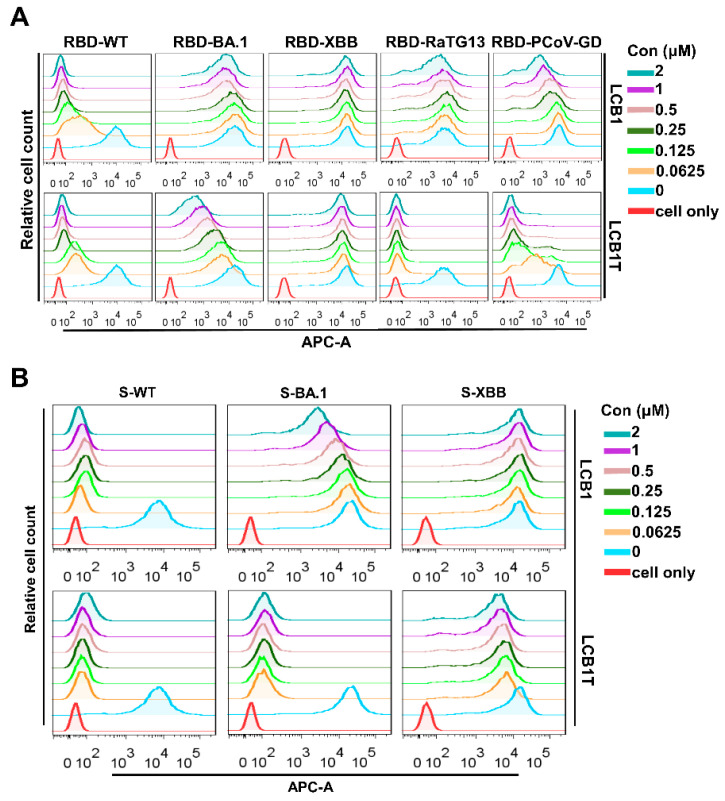
Blocking capacities of LCB1-derived recombinant protein and LCB1 inhibitor on the binding of RBD/S with ACE2. The blocking activities of recombinant protein and the LCB1 control on the binding of RBD (**A**) and S (**B**) with ACE2 on 293T/ACE2 cells, as determined through flow cytometry analysis.

To explore the mechanism underlying the multivalent LCB1 resistance, we firstly measured the blocking capacity of multivalent LCB1 on the binding of RBD proteins with ACE2 expressed on 293T/ACE2 cells, as determined using flow cytometry. Figure 4A illustrates that, as expected, the control LCB1 inhibitor could effectively obstruct the binding of RBD for SARS-CoV-2 WT. However, its capacity to block RBDs of BA.1, RaTG13, and PCoV-GD showed a significant reduction, and it exhibited no efficacy in the RBD binding of the XBB variant. In striking contrast, the trivalent LCB1 inhibitor exhibited robust efficacy in obstructing the binding of RBDs from WT, RaTG13, and PCoV-GD to cell surface ACE2. Nonetheless, its capacity to block the binding of RBD from BA.1 was slightly diminished, and for the RBD from XBB, it was completely eliminated.

Subsequently, we proceeded to assess the capability of the multivalent LCB1 inhibitor to obstruct the interaction between the spike protein and the cell surface ACE2. Consistent with the blocking activity on RBD binding above, as shown in Figure 4B, the binding of spike protein of WT was highly susceptible to inhibition by the trivalent LCB1 and template LCB1. In contrast, the binding of spike protein of XBB showed a pronounced resistance to their inhibitory effects. Differing from its previous activity in blocking RBD binding of the BA.1 variant on cell surface ACE2, the trivalent LCB1 exhibited an increased efficiency in hindering the binding of the spike protein in this context.

## 4. Discussion

In this study, we continued our efforts to create and characterize a novel RBM-targeted LCB1-based inhibitor and achieved some significant findings. First, we successfully engineered two multivalent LCB1 fusion proteins, denoted as LCB1T and LCB1T-Fc, which showcased significantly enhanced inhibitory potency against a wide array of SARS-CoV-2 strains compared to the LCB1 parental inhibitor in vitro. Second, in comparison to the D614G parental variant, the majority of emerging variants exhibited heightened susceptibility to inhibition by the two fusion proteins. However, among these variants, Delta, Lambda, and Omicron subvariants BQ.1.1, XBB, XBB.1.5, and EG.5.1 displayed varying degrees of resistance. Particularly, XBB, XBB.1.5, and EG.5.1 exhibited an exceptionally high level of resistance, with fold-changes greater than 208,000-fold, 6900-fold, or 700,000-fold, respectively. Third, with regard to the inhibition of the SARS-CoV-2-related coronaviruses, including bCoV-RaTG13, PCoV-GD, and PCoV-GX, the two fusion proteins also possessed substantially improved potency over the parental LCB1 inhibitor. Fourth, we further used flow cytometry to demonstrate the potent activity of LCB1T in obstructing the binding of RBD/S of susceptible strains with cell surface ACE2 receptor, and the high resistance in blocking the binding of RBD/S of resistant strains, implying the underlying susceptibility and resistance mechanism associated with multivalent LCB1 inhibitor. In conclusion, our study provides valuable information for understanding the antiviral profiling of LCB1-based multivalent proteins as well as the underlying mechanism, and confirms the feasibility of multimerization as a tool to enhance the activity of antiviral drugs, which will further guide the rational design of novel antivirals to combat the continuously evolving SARS-CoV-2. 

Following the confirmation of the RBD-ACE2 interaction as a crucial step for SARS-CoV-2 entry, there has been rapid progress in the development of prophylactic vaccines and therapeutic interventions targeting the RBD. Among therapeutics, the miniprotein-based LCB1 inhibitor, which specifically targets the spike RBD, was shown to be effective in neutralizing authentic virus in cell cultures at picomolar levels in vitro [12], highlighting its potential as a promising avenue for further exploration and development. The researchers subsequently constructed an optimized LCB1 derivative (LCB1v1.3) and an Fc domain-fused LCB1 version (LCB1-Fc) and demonstrated that they could confer high levels of protection and treatment against lethal SARS-CoV-2 historical and Alpha strain challenge in animal models [13]. Our group characterized the LCB1 inhibitor’s antiviral activities against the multiple pre-Omicron BA.1 variants and found that diverse variants could markedly impair its inhibitory potency [16]. By using a truncation and lipid modification strategy, we further generated several lipopeptide-based LCB1 inhibitors that were highly effective in inhibiting the LCB1-resistant variants [17]. To overcome the LCB1 resistance problem, other strategies including cyclization and multimerization have also been used to improve the antiviral activity of LCB1-derived inhibitors against divergent emerging SARS-CoV-2 variants [15,31,32,33]. Regrettably, the emerging virus variants still possessed mild resistance to the modified LCB1 inhibitors’ inhibition. In terms of neutralization potency, homo- and hetero-multimerization approaches exhibited the most powerful capabilities. Consistently, the neutralization capabilities of two homo-multimerized LCB1 constructs produced in this study were sharply enhanced. However, it was observed that the current circulating XBB, XBB.1.5, and EG.5.1 Omicron variants showed very high levels of resistance to the two multivalent LCB1 constructs. Very interestingly, XBB.1.5, a sublineage of the XBB variant, was more susceptible to their inhibition, which may be related to the F486 mutation in the spike RBM (Figure 2B). The F486 site in the RBM has been shown to play a pivotal role in facilitating the RBD-ACE2 interaction [34]. Indeed, the introduction of the F486P mutation, but not the F486S mutation, exhibited no detrimental impact on the interaction between S proteins and ACE2, but rather resulted in an augmentation of their binding affinity [35,36,37]. XBB.1.5 and XBB alone had a different RBD mutation, i.e., the F486 site mutation (F486P in XBB.1.5, and F486S in XBB). Therefore, we speculate that the sensitivity of XBB.1.5 to the two multivalent LCB1 inhibitors is potentially governed by the F486P mutation, which will need to be verified in our further investigation. EG.5.1 also has only one mutation in RBD compared to XBB.1.5, that is, the F456L mutation. It has been shown that the F456L mutation still escapes neutralization in serum after XBB.1.5 infection [38]. Consequently, the sensitivity of EG.5.1 to the two multivalent LCB1 inhibitors is drastically reduced, by at least about three-fold compared to XBB, which is inextricably associated with the F456L mutation. The BQ.1.1 and BF.7 variants, which are descendants of the BA.5 lineage, exhibited distinct responses to the multivalent LCB1 inhibitor. Notably, BQ.1.1 displayed a heightened level of resistance to the inhibitor, while BF.7 remained susceptible. The mechanism underpinning the resistance of BQ.1.1 to the inhibitor is possibly attributed to additional mutations at positions K444T and N460K. Studies have indicated that these two additional mutations, particularly the N460K mutation, conferred robust resistance to RBD-targeted neutralizing antibodies, in the context of BQ.1.1 [37,39]. In consideration of the attenuated resistance level of XBB.1.5 relative to the parental XBB variant and the context of the evolving pattern of SARS-CoV-2, it remains essential to continuously assess the effectiveness of such multivalent LCB1 inhibitors in response to the emergence of novel evolved variants.

The factors contributing to the above-mentioned coronavirus viral infections are by no means limited to RBD within the susceptible population. Viral infection is a complex and multifactorial process. To predict human coronavirus tropism, Singh M et al. analyzed 28 SARS-CoV-2 and coronavirus-associated receptors and factors (SCARF) in various healthy human tissues using single-cell transcriptomics [40]. These included ACE2 and transmembrane serine protease 2 (TMPRSS2), which have been identified as the primary receptors and key proteases for SARS-CoV-2 entry into cells. Basignin (BSG, also known as CD147) is a cell surface protein that has been shown to interact with S proteins in vitro and facilitate the entry of SARS-CoV and SARS-CoV-2 into Vero and 293T cells. Host factors, such as LY6E, can restrict the entry of SARS-CoV-2 into cells. Other factors that act after viral entry but relatively early in viral replication, such as TOP3B and MADP1 (ZCRB1), which may be expressed in a tissue-/cell-type-specific manner, are known to be critical for SARS-CoV-2 and SARS-CoV genome replication. And proteins are involved in the assembly and transport of a range of RNA viruses. These actors are critical for accomplishing the overall viral infection, and are the direction of our further research.

The LCB1 miniprotein acts as an ACE2 helix mimetic, displaying a proteomimetic nature. In contrast, multiple ACE2 receptor traps as inhibitors displayed the capacity to withstand mutations in the spike RBD [41,42,43,44,45,46], underscoring the stability of the interaction between ACE2 and all mutated RBD domains. Linsky and colleagues employed a de novo approach to design ACE2 decoys targeting RBD, which involved the fusion of minimal replication of ACE2’s binding interface with directed evolution of the supporting scaffold [21]. By means of sequence optimization, they successfully engineered a monomeric and highly stable protein decoy, termed CTC-445.2, with a remarkable nanomolar affinity for the RBD, as well as a noteworthy IC_50_ value. Importantly, CTC-445.2 showed robust resilience against SARS-CoV-2 RBD mutational evasion. And a subsequent trimerization conferred the decoy with a picomolar affinity for the RBD. Therefore, even in a de novo design strategy, certain residual components of the wild-type ACE2 interface proved instrumental in preserving the broad-spectrum neutralization efficacy against rapidly emerging variants. In the ongoing exploration of LCB1-derived inhibitors, sequence optimization or further increasing valency of LCB1 will offer a viable strategy for combating the newly evolved variants. Very recently, Liu et al. discovered that engineering two nanobodies targeting the RBD domain into dimeric, homo-trimeric, and decameric structures resulted in a substantial enhancement of their efficacy against Omicron subvariants when compared to their monomeric form. In particular, the decameric nanobodies displayed the most significant augmentation in neutralizing activity [47].

## Figures and Tables

**Table 1:** Inhibitory activity of LCB1-based inhibitors against divergent SARS-CoV-2 variants.

SARS-CoV-2	Mean IC_50_ ± SD (pM)					
	LCB1		LCB1T		LCB1T-Fc	
		Fold Change		Fold Change		Fold Change
D614G	94.83 ± 16.54	1	28.34 ± 1.33	1	29.05 ± 8.61	1
Alpha (B.1.1.7)	1377.34 ± 261.64	14.52	13.68 ± 0.98	0.48	15.83 ± 1.15	0.54
Beta (B.1.351)	514,742.27 ± 133,785.13	5428.05	3.29 ± 0.80	0.12	2.09 ± 0.14	0.07
Gamma (B.1.1.28)	269,999.99 ± 29,749.62	2847.20	0.63 ± 0.03	0.02	0.78 ± 0.024	0.03
Delta (B.1.617.2)	1303.39 ± 239.50	13.74	545.35 ± 33.10	19.24	790.00 ± 83.00	27.19
Lambda (C.37)	884.63 ± 222.62	9.33	210.23 ± 13.16	7.42	206.64 ± 24.76	7.11
Omicron variants						
BA.1	966,926.86 ± 43,184.25	10,196.42	3.22 ± 0.24	0.11	3.78 ± 0.63	0.13
BA.2	1,394,306.57 ± 268,000.37	14,703.22	5.08 ± 0.61	0.18	1.72 ± 0.38	0.06
BA.2.12.1	1,706,666.67 ± 321,601.61	17,997.12	3.28 ± 0.60	0.12	2.00 ± 0.44	0.07
BA.2.13	1,343,406.33 ± 11,023.91	14,166.47	2.77 ± 0.19	0.10	1.43 ± 0.10	0.05
BA.4/BA.5	5,514,841.85 ± 70,199.60	58,155.03	20.45 ± 4.91	0.72	3.79 ± 0.92	0.13
BF.7	3,855,474.45 ± 532.55	40,656.70	14.05 ± 4.79	0.50	6.18 ± 2.24	0.21
BQ.1.1	12,214,111.92 ± 4405.28	128,800.08	779.45 ± 65.99	27.50	267.88 ± 107.25	9.22
XBB	25,274,452.55 ± 2,835,889.33	266,523.81	9,026,246.43 ± 996,079.15	318,498.46	>6,045,949	>208,122.17
XBB.1.5 EG.5.1	>29,000,000 >72,803,460	>305,810.40 >767,726.04	382,280.70 ± 75,662.99 >24,028,654	13,489.06 >847,870.64	177,785.17 ± 13,059.25 >20,887,539	6119.97 >719,020.28

## Data Availability

All data are fully available without restriction.

## References

[1] Faraone J.N., Qu P., Evans J.P., Zheng Y.M., Carlin C., Anghelina M., Stevens P., Fernandez S., Jones D., Lozanski G. (2023). Neutralization escape of Omicron XBB, BR.2, and BA.2.3.20 subvariants. Cell Rep. Med..

[2] Feikin D.R., Higdon M.M., Andrews N., Collie S., Deloria Knoll M., Kwong J.C., Link-Gelles R., Pilishvili T., Patel M.K. (2023). Assessing COVID-19 vaccine effectiveness against Omicron subvariants: Report from a meeting of the World Health Organization. Vaccine.

[3] Tian D., Nie W., Sun Y., Ye Q. (2022). The Epidemiological Features of the SARS-CoV-2 Omicron Subvariant BA.5 and Its Evasion of the Neutralizing Activity of Vaccination and Prior Infection. Vaccines.

[4] Xiang T., Wang J., Zheng X. (2022). The humoral and cellular immune evasion of SARS-CoV-2 Omicron and sub-lineages. Virol. Sin..

[5] Yao L., Zhu K.L., Jiang X.L., Wang X.J., Zhan B.D., Gao H.X., Geng X.Y., Duan L.J., Dai E.H., Ma M.J. (2022). Omicron subvariants escape antibodies elicited by vaccination and BA.2.2 infection. Lancet Infect. Dis..

[6] Ou X., Liu Y., Lei X., Li P., Mi D., Ren L., Guo L., Guo R., Chen T., Hu J. (2020). Characterization of spike glycoprotein of SARS-CoV-2 on virus entry and its immune cross-reactivity with SARS-CoV. Nat. Commun..

[7] Tai W., He L., Zhang X., Pu J., Voronin D., Jiang S., Zhou Y., Du L. (2020). Characterization of the receptor-binding domain (RBD) of 2019 novel coronavirus: Implication for development of RBD protein as a viral attachment inhibitor and vaccine. Cell. Mol. Immunol..

[8] Nguyen H.T., Zhang S., Wang Q., Anang S., Wang J., Ding H., Kappes J.C., Sodroski J. (2021). Spike glycoprotein and host cell determinants of SARS-CoV-2 entry and cytopathic effects. J. Virol..

[9] Shang J., Ye G., Shi K., Wan Y., Luo C., Aihara H., Geng Q., Auerbach A., Li F. (2020). Structural basis of receptor recognition by SARS-CoV-2. Nature.

[10] Barton M.I., MacGowan S.A., Kutuzov M.A., Dushek O., Barton G.J., van der Merwe P.A. (2021). Effects of common mutations in the SARS-CoV-2 Spike RBD and its ligand, the human ACE2 receptor on binding affinity and kinetics. Elife.

[11] Wrapp D., Wang N.S., Corbett K.S., Goldsmith J.A., Hsieh C.L., Abiona O., Graham B.S., McLellan J.S. (2020). Cryo-EM structure of the 2019-nCoV spike in the prefusion conformation. Science.

[12] Cao L., Goreshnik I., Coventry B., Case J.B., Miller L., Kozodoy L., Chen R.E., Carter L., Walls A.C., Park Y.J. (2020). De novo design of picomolar SARS-CoV-2 miniprotein inhibitors. Science.

[13] Case J.B., Chen R.E., Cao L., Ying B., Winkler E.S., Johnson M., Goreshnik I., Pham M.N., Shrihari S., Kafai N.M. (2021). Ultrapotent miniproteins targeting the SARS-CoV-2 receptor-binding domain protect against infection and disease. Cell Host Microbe.

[14] Javanmardi K., Chou C.W., Terrace C.I., Annapareddy A., Kaoud T.S., Guo Q., Lutgens J., Zorkic H., Horton A.P., Gardner E.C. (2021). Rapid characterization of spike variants via mammalian cell surface display. Mol. Cell.

[15] Hunt A.C., Case J.B., Park Y.J., Cao L., Wu K., Walls A.C., Liu Z., Bowen J.E., Yeh H.W., Saini S. (2022). Multivalent designed proteins neutralize SARS-CoV-2 variants of concern and confer protection against infection in mice. Sci. Transl. Med..

[16] Wu T., Zhu Y., Liu N., Hu Y., Chong H., He Y. (2022). Resistance profile and mechanism of severe acute respiratory syndrome coronavirus-2 variants to LCB1 inhibitor targeting the spike receptor-binding motif. Front. Microbiol..

[17] Zhu Y., Li M., Liu N., Wu T., Han X., Zhao G., He Y. (2023). Development of highly effective LCB1-based lipopeptides targeting the spike receptor-binding motif of SARS-CoV-2. Antivir. Res..

[18] Silverman J., Liu Q., Bakker A., To W., Duguay A., Alba B.M., Smith R., Rivas A., Li P., Le H. (2005). Multivalent avimer proteins evolved by exon shuffling of a family of human receptor domains. Nat. Biotechnol..

[19] Detalle L., Stohr T., Palomo C., Piedra P.A., Gilbert B.E., Mas V., Millar A., Power U.F., Stortelers C., Allosery K. (2016). Generation and Characterization of ALX-0171, a Potent Novel Therapeutic Nanobody for the Treatment of Respiratory Syncytial Virus Infection. Antimicrob. Agents Chemother..

[20] Strauch E.M., Bernard S.M., La D., Bohn A.J., Lee P.S., Anderson C.E., Nieusma T., Holstein C.A., Garcia N.K., Hooper K.A. (2017). Computational design of trimeric influenza-neutralizing proteins targeting the hemagglutinin receptor binding site. Nat. Biotechnol..

[21] Linsky T.W., Vergara R., Codina N., Nelson J.W., Walker M.J., Su W., Barnes C.O., Hsiang T.Y., Esser-Nobis K., Yu K. (2020). De novo design of potent and resilient hACE2 decoys to neutralize SARS-CoV-2. Science.

[22] Schoof M., Faust B., Saunders R.A., Sangwan S., Rezelj V., Hoppe N., Boone M., Billesbolle C.B., Puchades C., Azumaya C.M. (2020). An ultrapotent synthetic nanobody neutralizes SARS-CoV-2 by stabilizing inactive Spike. Science.

[23] Bracken C.J., Lim S.A., Solomon P., Rettko N.J., Nguyen D.P., Zha B.S., Schaefer K., Byrnes J.R., Zhou J., Lui I. (2021). Bi-paratopic and multivalent VH domains block ACE2 binding and neutralize SARS-CoV-2. Nat. Chem. Biol..

[24] Koenig P.A., Das H., Liu H., Kummerer B.M., Gohr F.N., Jenster L.M., Schiffelers L.D.J., Tesfamariam Y.M., Uchima M., Wuerth J.D. (2021). Structure-guided multivalent nanobodies block SARS-CoV-2 infection and suppress mutational escape. Science.

[25] Rothenberger S., Hurdiss D.L., Walser M., Malvezzi F., Mayor J., Ryter S., Moreno H., Liechti N., Bosshart A., Iss C. (2022). The trispecific DARPin ensovibep inhibits diverse SARS-CoV-2 variants. Nat. Biotechnol..

[26] Li M.X., Ren Y.F., Aw Z.Q., Chen B., Yang Z.Q., Lei Y.Q., Cheng L., Liang Q.T., Hong J.X., Yang Y.L. (2022). Broadly neutralizing and protective nanobodies against SARS-CoV-2 Omicron subvariants BA.1, BA.2, and BA.4/5 and diverse sarbecoviruses. Nat. Commun..

[27] Zhang Y., Zhang L., Wu J.J., Yu Y.L., Liu S., Li T., Li Q.Q., Ding R.X., Wang H.X., Nie J.H. (2022). A second functional furin site in the SARS-CoV-2 spike protein. Emerg. Microbes Infec..

[28] Zhu Y.M., Dong X.J., Liu N.A., Wu T., Chong H.H., Lei X.B., Ren L.L., Wang J.W., He Y.X. (2022). SARS-CoV-2 fusion-inhibitory lipopeptides maintain high potency against divergent variants of concern including Omicron. Emerg. Microbes Infec..

[29] Jin H.L., Cheng L., Gong Y.N., Zhu Y.M., Chong H.H., Zhang Z., He Y.X. (2023). Design of a bifunctional pan-sarbecovirus entry inhibitor targeting the cell receptor and viral fusion protein. J. Virol..

[30] Zhou B., Cheng L., Song S., Guo H., Shen S., Wang H., Ge X., Liu L., Ju B., Zhang Z. (2022). Identification and application of a pair of noncompeting monoclonal antibodies broadly binding to the nucleocapsid proteins of SARS-CoV-2 variants including Omicron. Virol. J..

[31] Weissenborn L., Richel E., Huseman H., Welzer J., Beck S., Schafer S., Sticht H., Uberla K., Eichler J. (2022). Smaller, Stronger, More Stable: Peptide Variants of a SARS-CoV-2 Neutralizing Miniprotein. Int. J. Mol. Sci..

[32] Chattaraj R., Kim C.Y., Lee D., Hammer D.A. (2022). Recombinant Protein Micelles to Block Transduction by SARS-CoV-2 Pseudovirus. ACS Nano.

[33] Llewellyn G.N., Chen H.Y., Rogers G.L., Huang X., Sell P.J., Henley J.E., Cannon P.M. (2023). Comparison of SARS-CoV-2 entry inhibitors based on ACE2 receptor or engineered Spike-binding peptides. J. Virol..

[34] Cao Y., Jian F., Wang J., Yu Y., Song W., Yisimayi A., Wang J., An R., Chen X., Zhang N. (2023). Imprinted SARS-CoV-2 humoral immunity induces convergent Omicron RBD evolution. Nature.

[35] Callaway E. (2023). Coronavirus variant XBB.1.5 rises in the United States - is it a global threat?. Nature.

[36] Graham F. (2023). Daily briefing: Is subvariant XBB.1.5 a global threat?. Nature.

[37] Qu P., Evans J.P., Faraone J.N., Zheng Y.M., Carlin C., Anghelina M., Stevens P., Fernandez S., Jones D., Lozanski G. (2023). Enhanced neutralization resistance of SARS-CoV-2 Omicron subvariants BQ.1, BQ.1.1, BA.4.6, BF.7, and BA.2.75.2. Cell Host Microbe.

[38] Parums D.V. (2023). Editorial: A Rapid Global Increase in COVID-19 is Due to the Emergence of the EG.5 (Eris) Subvariant of Omicron SARS-CoV-2. Med. Sci. Monit..

[39] Wang Q., Iketani S., Li Z., Liu L., Guo Y., Huang Y., Bowen A.D., Liu M., Wang M., Yu J. (2023). Alarming antibody evasion properties of rising SARS-CoV-2 BQ and XBB subvariants. Cell.

[40] Singh M., Bansal V., Feschotte C. (2020). A Single-Cell RNA Expression Map of Human Coronavirus Entry Factors. Cell Rep..

[41] Chan K.K., Tan T.J.C., Narayanan K.K., Procko E. (2021). An engineered decoy receptor for SARS-CoV-2 broadly binds protein S sequence variants. Sci. Adv..

[42] Ferrari M., Mekkaoui L., Ilca F.T., Akbar Z., Bughda R., Lamb K., Ward K., Parekh F., Karattil R., Allen C. (2021). Characterization of a Novel ACE2-Based Therapeutic with Enhanced Rather than Reduced Activity against SARS-CoV-2 Variants. J. Virol..

[43] Higuchi Y., Suzuki T., Arimori T., Ikemura N., Mihara E., Kirita Y., Ohgitani E., Mazda O., Motooka D., Nakamura S. (2021). Engineered ACE2 receptor therapy overcomes mutational escape of SARS-CoV-2. Nat. Commun..

[44] Arimori T., Ikemura N., Okamoto T., Takagi J., Standley D.M., Hoshino A. (2022). Engineering ACE2 decoy receptors to combat viral escapability. Trends Pharmacol. Sci..

[45] Wang B., Zhao J., Liu S., Feng J., Luo Y., He X., Wang Y., Ge F., Wang J., Ye B. (2022). ACE2 decoy receptor generated by high-throughput saturation mutagenesis efficiently neutralizes SARS-CoV-2 and its prevalent variants. Emerg. Microbes Infect..

[46] Zhang H., Hu B., Lv P., Liu Y., Guo M., Wu Z., Zhou K., Dai M., Yu X., Liu Z. (2022). An ACE2-Based Decoy Inhibitor Effectively Neutralizes SARS-CoV-2 Omicron BA.5 Variant. Viruses.

[47] Liu H., Wu L., Liu B., Xu K., Lei W., Deng J., Rong X., Du P., Wang L., Wang D. (2023). Two pan-SARS-CoV-2 nanobodies and their multivalent derivatives effectively prevent Omicron infections in mice. Cell Rep. Med..

